# Search for Disease-Specific Genetic Markers Originated from the Vitamin D Binding Protein Gene Polymorphisms in the Multiple Sclerosis Cohort in the Latvian Population

**DOI:** 10.3390/ijms26062555

**Published:** 2025-03-12

**Authors:** Jolanta Kalnina, Ilva Trapina, Samanta Plavina, Elina Leonova, Jegors Paramonovs, Nikolajs Sjakste, Natalia Paramonova

**Affiliations:** Genomic and Bioinformatic Laboratory, The Department of Pharmaceutical Sciences, Faculty of Medicine and Life Sciences, the University of Latvia, LV-1004 Riga, Latvia; jolanta@ljmc.lv (J.K.); samanta.plavina@lu.lv (S.P.); elina.leonova@lu.lv (E.L.); jegors.paramonovs@lu.lv (J.P.); nikolajs.sjakste@lu.lv (N.S.); natalia.paramonova@lu.lv (N.P.)

**Keywords:** multiple sclerosis, rs4588 (Thr436Lys), rs7041 (Asp432Glu), single Nucleotide Polymorphisms (SNPs), Vitamin D Binding Protein (VDBP), 25-hydroxyvitamin D (25(OH)D)

## Abstract

Vitamin D is crucial for immune regulation, and its deficiency is linked to multiple sclerosis (MS). The GC gene encodes Vitamin D Binding Protein (VDBP), which regulates vitamin D transport and bioavailability. This study examines the association of *GC* polymorphisms (rs7041, rs4588) with MS susceptibility and their impact on 25-hydroxyvitamin D [25(OH)D] levels in a Latvian cohort. This case–control study included 296 MS patients and 253 healthy controls. Genotyping of rs7041 and rs4588 was conducted using restriction fragment length polymorphism analysis and validated by Sanger sequencing. Plasma 25(OH)D levels were measured in 131 MS patients using an enzyme-linked immunosorbent assay. Statistical analysis included Hardy–Weinberg equilibrium testing, Fisher’s exact test, allelic and genotypic frequency comparisons to assess MS risk, and the Kruskal–Wallis test for 25(OH)D level differences among genotypes. Our findings indicate that the rare rs7041-T and rs4588-A alleles, along with their corresponding haplotypes, exhibit a protective effect against MS (*p* < 0.001; OR = 0.65 for rs4588-A; *p* < 0.01; OR = 0.70 for rs7041-T). Conversely, the common rs7041-G and rs4588-C alleles were associated with an increased MS risk (*p* < 0.05). Individuals with the Gc1F/1F isotype had the highest average 25(OH)D levels (29.31 ng/mL), while Gc1S/2 carriers had the lowest (21.53 ng/mL). Our results indicate that *GC* polymorphisms may influence the susceptibility of Latvians to MS and vitamin D status.

## 1. Introduction

Multiple sclerosis (MS) is an inflammatory demyelinating disease of the central nervous system with a rather complex etiology. According to the Multiple Sclerosis Atlas, the global prevalence of MS rose to 2.9 million in 2023, up from 2.8 million in 2020. The prevalence of MS in Europe shows significant variation, ranging from 32 to 300 cases per 100,000 people [1]. North-eastern Europe, the Baltic region, including Latvia, is among the regions with a high incidence of multiple sclerosis [1]. Latvia, with a population of approximately 1.8 million, has around 2035 confirmed MS cases, resulting in an incidence rate of 107 per 100,000 people [1,2]. Given the significant disease burden, deeper exploration of MS-related genetic and environmental interactions in this population is critical.

Vitamin D deficiency is a global health concern, linked not only to bone mineralization defects but also to a range of chronic, autoimmune, and infectious diseases, including cancer, cardiovascular disease, diabetes, rheumatoid arthritis, tuberculosis, and multiple sclerosis [3,4,5,6,7,8]. Numerous studies have suggested that sunlight exposure, which is essential for vitamin D synthesis, may have a protective effect against MS [8,9]. Conversely, decreased sunlight exposure at higher latitudes, such as those found in the Baltic region, may lead to vitamin D deficiency, potentially contributing to the disease [9,10].

Vitamin D is typically determined by plasma 25-hydroxyvitamin D (25(OH)D) concentration, an integrated marker of different sources of vitamin D supply [11,12]. Importantly, low serum levels of 25-hydroxyvitamin D (25(OH)D), the primary circulating form of vitamin D, have been consistently associated with an increased risk of MS [8]. Furthermore, genetic variations in genes involved in vitamin D metabolism have been identified as potential contributors to the onset and progression of the disease [13].

The main transporter of vitamin D metabolites in the circulation is the multifunctional plasma Gc protein, also known as the group component, Gc globulin, vitamin D binding protein, or VDBP [14,15,16,17]. The functions of VDBP are to bind 25(OH)D to prolong its half-life in circulation and transport vitamin D metabolites to tissues to facilitate vitamin D physiological roles, such as cell proliferation, apoptosis, and differentiation [14,18,19,20]. The protein VDBP is encoded by the GC gene (identification no. in the NCBI database—ID2638), located on the long arm of chromosome 4 (4q12-q13) and localized in the genome at position GRCh38:CM000666.2 [18,21].

Genetic variations in the GC gene can lead to significant differences in VDBP serum levels and binding affinity, influencing individual vitamin D status and potentially affecting health outcomes [22,23,24]. Among the various *GC* polymorphisms, rs7041 (Asp432Glu) and rs4588 (Thr436Lys) are of particular interest due to their strong impact on VDBP structure and function. These two missense variants are located in exon 11 of the GC gene and determine the three major VDBP isoforms, which directly correspond to distinct protein phenotypes. According to the traditional nomenclature, they are classified as follows: (1) Gc1F—fast protein isotype; (2) Gc1S—slow; and (3) Gc2 [18,20]. The designations in the name of the protein originate from the detected differences in their movements in gel electrophoresis using the isoelectric focusing method; namely, the Gc1F isoform moved faster in the gel than the Gc1S isoform, thus indicating their structural differences [25]. The protein configurations of these isotypes differ in their degree of binding affinity to 25(OH)D, which leads to a change in serum 25(OH)D concentration [26] and may correlate with an increased risk of certain health conditions [27,28,29]. 

Considering that the alleles of the above SNPs are in high linkage disequilibrium (LD) in different populations, it is possible to identify three haplotypes by their combinations in humans: (1) Gc1F (rs7041-T/rs4588-C), (2) Gc1S (rs7041-G/rs4588-C) and (3) Gc2 (rs7041-T/rs4588-A), and six genotypes by which possible types of circulating VDBP protein isotypes can be determined (Gc1F/1F, Gc1F/1S, Gc1F/2, Gc1S/1S, Gc1S/2, Gc2/2). Thus, an individual has one or two different forms of VDBP in their circulation, depending on whether it has a homozygous (Gc1F/1F, Gc1S/1S, Gc2/2) or heterozygous (Gc1F/2, Gc1F/1S, Gc1S/2) genotype [21,26].

The selection of rs7041 and rs4588 for this study was based on their well-documented role in vitamin D metabolism and their potential impact on MS susceptibility [8,20,23,25]. Furthermore, these polymorphisms have been studied in various populations, with divergent results highlighting the importance of population-specific genetic factors in disease susceptibility [20,30,31]. For instance, while some studies have found no association between these SNPs and MS risk in certain populations [32,33,34], others have reported significant associations, particularly in populations with high prevalence rates of vitamin D deficiency [35]. Divergent results have been published across populations describing either very similar or several-fold differences in affinity between the 25(OH)D and VDBP isoforms [31,32]. VDBP and its interaction with 25(OH)D may be an essential factor in our interpretation of the physiological effects of vitamin D. This variability calls for population-specific studies to better understand the genetic factors underlying MS.

The Latvian population provides a unique opportunity to study the interplay between genetic and environmental factors in MS. Latvia’s geographical location at a high latitude result in limited sunlight exposure, particularly during the winter months, which may contribute to widespread vitamin D deficiency [1]. Additionally, the Latvian population has a distinct genetic background shaped by historical and demographic factors, which may influence the prevalence of specific genetic variants and their association with disease [36]. This unique combination of environmental and genetic factors makes the Latvian population an ideal cohort for investigating the role of GC gene polymorphisms in MS susceptibility.

In this study, we aimed to investigate the association between GC gene polymorphisms (rs7041 and rs4588) and MS susceptibility in the Latvian population, with a particular focus on their impact on plasma 25(OH)D levels. By examining these specific SNPs, we sought to elucidate the role of vitamin D metabolism in MS pathogenesis and to identify potential genetic markers for disease risk in this population. Our findings contribute to the growing body of evidence on the genetic basis of MS and highlight the importance of population-specific studies in understanding the complex interplay between genetics, environment, and disease.

## 2. Results

### 2.1. Polymorphisms Discovery and Genetic Diversity

In the current study, we have genotyped the rs4588 and rs7041 in 296 MS patients and 253 healthy individuals.

In both MS and in a control group in Latvians, the rs4588 and rs7041 genotyping call rate was 100%, and the markers were found in the HWE (*p*> 0.05). Both GC gene SNPs showed a strong association in the LD patterns of both cohorts (Table 1). It should be noted that higher LD scores were found in the MS group (D′ = 0.99; r^2^ = 0.60; *p* < 0.00001) compared to the control group (D′ = 0.80; r^2^ = 0.39; *p* < 0.0001).

Both investigated loci showed statistically significant protective MS-related effect for rare alleles rs4588 (A): *p* < 0.001; OR = 0.65, 95% confidence interval (CI) [0.51–0.83] and rs7041 (T): *p* < 0.01; OR = 0.70, 95% CI [0.54–0.90], additive model. Homozygotes with rare alleles included have been identified as clinical protective factors (rs4588, *p* < 0.001, OR= 0.39, 95% CI [0.24–0.64], and rs7041, *p* < 0.01, OR = 0.40, 95% CI [0.21–0.76]), additive model (Table 2). Additionally, genotypes including common alleles as MS-risk-related factors (*p*< 0.05) were identified in the disease cohort for rs4588 (CC), OR = 1.50, 95% CI [1.02–2.16]), and rs7041 (GG), OR = 1.52, 95% CI [1.08–2.15]) multiplicative statistic model). For heterozygotes of both loci, the minimum protective effect in relation to the disease was determined using an additive statistic model (*p*< 0.05; Table 2).

### 2.2. Multi-Locus Genotypes, DBP Haplotypes, and Gc Isotype Variant Distribution

The data obtained from multi-locus analysis of the studied SNPs are presented in Table 3.

In the Latvian population, the most frequent (reference) was haplotype H1 (G-C)/coding 1S protein isotype, present in 58.61% of cases and 45.26% of controls, respectively. In the current study, this haplotype was associated with a high risk of multiple sclerosis. (*p* < 0.00001, OR_M_ = 1.72, 95% CI 1.36–2.19). Accordingly, the remaining haplotypes showed a protective effect of varying significance: for H2, coding 1F protein isotype, it was *p* < 0.01; for H3 and rarest haplotype H4, coding the same protein isotype 2, it was on the border of statistical significance (*p* = 0.078) and *p* < 0.0001, respectively.

The second most frequent, constituting 29.90% and 34.78% in each group, was haplotype H3 (T-A) coding protein isotype 2. The haplotype T-C, coding protein isotype 1F, was found in 11.32% of MS cases and 16.80% of controls. Also in the disease group, one case of detection of the rare haplotype G-A was noted.

Nine (9) two-locus genotypes rs7041-rs4588 were identified in a case–control study in the Latvian population. Five (G1-G5) were the most common, with a frequency of more than 5%; the remaining genotypes (G6-G9) were rare in the Latvian population. In addition, G8 and G9 were identified only in the control group of participants in the current study.

The Gc1S/2 was the most represented genotype in MS and control groups (40.54 and 34.66%, respectively). The G2 genotype GG-CC, with rare alleles included at both loci and coding Gc1S/1S protein isotype, was represented by 31.42% in cases and 19.92% in the controls groups and showed a significant disease-related risk effect in Latvians (*p* < 0.01; OR_M_ = 1.85, 95%CI [1.25–2.76]). All genotypes from the present distribution, including a rs4588- A rare allele, showed, on the contrary, a potential protective effect: G3 (TT-CA), represented by Gc1F/2 protein isotype variation (*p* < 0.01; OR_A_ = 0.42, 95%CI [0.23–0.77] and OR_M_ = 0.45, 95%CI [0.26–0.80], according to additive and multiplicative model, respectively); the rare genotype G7/Gc1S/2 (GG-CA), presented only in 1 case in the MS cohort and 7 cases in the controls (*p* < 0.05); the rarest genotypes G8 (TG-AA) and G9 (GG-AA), representing protein isotype variations that encode the same protein isoform Gc2/2, but with only one allele change in the genotype, were identified in our population cohort only in the control group (three cases each).

### 2.3. Comparison of Biochemical Data and z/Gc Isotype Distribution in MS Cohort

No statistically significant difference was found between average 25(OH)D levels in MS patients with different rs7041-rs4588 genotypes and related DBP isotypes (Table 4). However, it was to be the highest (29.31 ng/mL) in relation to others in two representatives of the rarest genotype TT-CC/DBP isotype Gc1F/1F variant, and the lowest (21.53 ng/mL)—in representatives of references genotype TG-CA, coding DBP isotype Gc1S/2 and constituting 45% of the experimental MS cohort.

## 3. Discussion

In this study, we focused on the key missense SNPs, rs7041 (Asp432Glu) and rs4588 (Thr436Lys), located on the 11th exon of the group-specific component (GC) gene, which is part of the vitamin D metabolic pathway and is believed to play a significant role in the development of multiple sclerosis. We genotyped these SNPs in 296 MS patients and 253 healthy controls to examine their potential association with multiple sclerosis. Our findings showed a statistically significant protective effect for the rare alleles and homozygotes with rare alleles, particularly for rs7041 (TT) and rs4588 (AA). We also observed a minor protective effect for heterozygotes at both loci. Conversely, homozygous genotypes for the common alleles, rs7041 (GG) and rs4588 (CC), were identified as risk factors for developing MS.

A similar associative study was conducted by us earlier in a cohort of patients with bronchial asthma in Latvians; rs7041 (GG) and rs4588 (CC) were found to be associated with a possible BA risk-increasing effect in Latvians (data not published).

**Figure 1:**
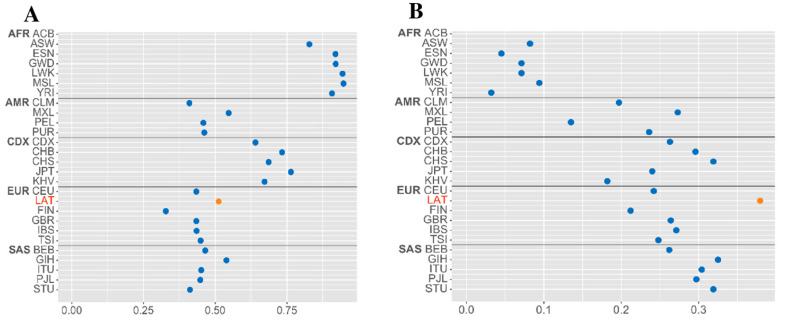
Minor allele frequency (MAF) distribution of the studied (**A**) rs7041 and (**B**) rs4588 among populations. MAF in Latvians (current study) is marked in orange. MAF for each population listed on the y-axis is marked in blue. Subpopulation groups: EAS—East Asia; EUR—European; AFR—African; AMR—Mixed American; SAS—South Asia. (According to the Ensembl database (1000 Genomes Project data, https://www.internationalgenome.org/category/ensembl/ accessed on 30 June 2023).

Comparing the frequencies of minor alleles (MAF) of the studied rs7041 and rs4588 (Figure 1) among populations, it was stated that MAF rs4588 (A) (0.38), referring to the cohort of the Latvian population, prevailed over the general frequency range determined in European populations (0.21–0.27) and the total MAF (0.27) among populations. The rs7041 (T) MAF value (0.52) was determined to be insignificantly prevailing in relation to the European population (0.43) and total MAF (0.45).

Both GC gene SNPs showed strong association in the LD patterns of both cohorts, cases, and controls; however, LD scores were found to predominate in the MS group (D′ = 0.99; r^2^ = 0.60) compared to the control group (D′ = 0.80; r^2^ = 0.39). These SNPs are also characterized by strong LD in other populations [21,22,37].

Differences in the frequencies of the above SNPs’ genetic variations at racial and interpopulation levels have been reported. The dominant allele in rs7041 (G) in Caucasians is the minor allele in those with African heritage [21]. 

The difference in minor allele frequencies between populations can lead to varying associations between genetic markers and multiple sclerosis, as the prevalence of specific alleles may differ across populations due to historical, demographic, and evolutionary factors [36]. These population-specific differences can impact on the statistical power and effect sizes observed in genetic studies, highlighting the importance of considering diverse populations for a comprehensive understanding of the genetic basis of multiple sclerosis.

For locus rs4588, allele-level associations varied across regions—within the European region, including this study, the minor allele frequency (MAF) was higher in controls (0.38) compared to the disease cohort (0.30) (Table 2); in the Egyptian population, this difference was determined to be statistically significant (*p* = 0.02) [38]; the rare allele A of rs4588 was identified as a significant risk factor of childhood asthma in the East Asian region [39].

In the present study, the rare allele rs4588-A, compared to the disease cohort in the Latvian population compared to the European cohort, was identified as having a protective effect associated with MS (*p* < 0.0001), as well as being present in different genotype variants: (TT-CA), coding Gc1F/2 protein isotype (*p* < 0.01); and the rare protein isotype Gc1S/2 (GG-CA), presented only in one case in the MS cohort and in seven cases in the controls (*p* < 0.05). Interestingly, the rarest genotypes (TG-AA) and (GG-AA), which encode the same protein isoform Gc2/2, but with only one allele change in the genotype, were identified in our population cohort (three cases each, Table 3).

Accordingly, the multi-locus analysis of our data was validated at the level of haplotype associations. In the current study, the most frequent (reference) haplotype was H1 (rs7041G-rs4588 C), formed by two common alleles previously found in association with MS and encoding the Gc1S protein isotype. The frequencies obtained by us correspond to the 1s isotype distribution in European populations [40]. In the current study, it was found associated with a high risk of multiple sclerosis (*p* < 0.00001, Table 3). Interestingly, for haplotype 1s and genotype Gc1S/1S, we determined a statistically reliable association with asthma in Latvians—specifically, a risk-increasing effect of BA; accordingly, haplotype Gc2 is determined as a protective variant (data not published).

The remaining haplotypes, coding Gc1F and Gc2 protein isotypes, showed a protective effect of varying significance. Moreover, one of these protective haplotypes, H3 (T-A) coding protein isotype Gc2, was identified as the second most frequent, constituting 29.90% and 34.78% in cases and controls, respectively. In the Latvian population cohort, cases of finding a rare haplotype, encoding the same protein isotype Gc2, with a protective effect, related to MS (*p* < 0.0001), were also stated. The haplotype T-C, coding protein isotype Gc1F, was found only in 11.32% of MS cases and 16.80% of controls in Latvians. In turn, the distribution of these DPB phenotypes in the Italian disease cohort was analyzed, resulting in no differences found between MS patients in the disease cohort [41].

In the present study, we did not detect a statistically significant difference between average 25(OH)D levels in MS patients with different rs7041-rs4588 genotypes and related DBP isotypes (Table 4). However, the following trend was noticed; it was to be the highest (29.31 ng/mL) in two representatives of the genotype TT-CC/Gc1F/1F isotype variant, rarest in Latvians; in turn, the lowest level (21.53 ng/mL), related to the TG-CA genotype encoding the DBP Gc1S/Gc2 isotype, was identified and constituting 45% of the experimental MS cohort. These results are consistent with previously published data: Gc1S haplotype and Gc1S/1S genotype have been associated with 25-(OH) sufficiency, while Gc1F/1S, Gc1F/2, Gc1S/2, Gc2/2, and Gc2/2 genotypes have been associated with 25-(OH) deficiency in the Jordanian population [42]. In a Han Chinese population, the Gc2 haplotype, or more exactly rs4588A was a risk factor for low vitamin D status [26].

Inconsistent data on associations of the polymorphisms under study with vitamin D deficiency and multiple sclerosis have been published in various sources related to studies in various populations.

There is a lack of association between 25-hydroxyvitamin D and MS risk among those with African heritage and Hispanics [33]. Xin Zhang et al. provide evidence that VDBP rs7041 and 4588 polymorphisms may not be associated with an increased risk of multiple sclerosis in the meta-analysis [34]. A homozygous recessive genotype for rs7041 has been found to be associated with insufficient 25(OH)D levels and the risk of multiple sclerosis in the Syrian population [35]. Significant associations with MS for both rs7041 and rs4588 loci were found in the current study, in the Latvian population.

Other studies that have looked at differences between different *GC* genotypes may have been confounded by other genetic factors (e.g., influencing skin type), ethnicity, and environmental factors, particularly UVB exposure, lifestyle, and different dietary habits that may have influenced plasma 25(OH)D. Langer-Gould et al. suggested that these differences cannot be explained by racial/ethnic variations in bioavailable vitamin D [33].

### 3.1. Study Limitations

Calcitriol metabolism is controlled by a complex interplay of genetic, dietary, and environmental factors, making it difficult to obtain standardized data on 25(OH)D levels. This may partly explain the lack of associations in our study. Drug therapy and vitamin D supplementation may influence VDBP levels and vitamin D bioavailability, potentially confounding genotype-25(OH)D associations. Medications like corticosteroids or immunomodulators could alter VDBP expression [43], while long-term supplementation may affect vitamin D homeostasis, warranting further investigation in the Latvian MS cohort [44]. Although serum sampling for vitamin D measurements was conducted in study participants over a period of one month, it excluded seasonal fluctuations in weather, the amount of melanin pigment in the skin, and lifestyle factors such as exposure time. Sunlight and diet can also significantly affect 25(OH)D levels [45]. Information was collected on study participants’ vitamin D supplementation habits over the past six months, but these data were not available for all study participants, creating an unresolved confounding factor in the data that could affect the outcome of the data analysis.

Epigenetic mechanisms, such as DNA methylation, histone modifications, and non-coding RNAs, may regulate GC gene expression and vitamin D metabolism [46]. DNA methylation of the GC gene could influence VDBP levels [47], while histone modifications might alter chromatin accessibility [48]. Additionally, miRNAs could post-transcriptionally regulate *GC* mRNA [49,50]. Environmental factors like UV exposure and diet may drive these epigenetic changes, potentially impacting vitamin D bioavailability and MS susceptibility [51]. Further research into these mechanisms could enhance understanding of the genetic-environmental interplay in vitamin D metabolism.

Another analytical limitation of the current study is the uneven group sizes of different DBP isotypes in our sample. An additional limitation is the small number of biochemical markers available for analysis. Analytes such as DBP concentration, free 25(OH)D, albumin, and calculated free 25(OH)D would have provided some additional mechanistic insight into the role of DBP genotype on vitamin D metabolism and these should be included in future studies.

### 3.2. Clinical Implications

The findings of this study have significant clinical implications for MS management and prevention, particularly in high-prevalence populations like Latvia. The protective effects of rare alleles (rs7041-T and rs4588-A) and the risk associated with common alleles (rs7041-G and rs4588-C) in the GC gene suggest potential for personalized risk assessment and targeted interventions. Individuals with high-risk genotypes may benefit from closer monitoring and tailored vitamin D supplementation to optimize serum levels and reduce disease risk. The observed differences in 25(OH)D levels among VDBP isotype carriers (e.g., higher in Gc1F/1F, lower in Gc1S/2) highlight the need for genotype-specific therapeutic strategies. These results suggest that genetic screening could be included in early intervention programs to help create personalized prevention plans, such as lifestyle changes and supplements. Additional studies in larger, diverse populations are necessary to validate these results and explore *GC* genotyping as a tool for personalized MS management.

## 4. Materials and Methods

### 4.1. Study Population and Sample Recruitment

This case–control study included 296 MS patients (of whom 288 had detailed clinical and disease history, see Table S1) and 253 healthy individuals from Latvia. MS patients were recruited from the Latvian Maritime Medicine Centre, Vecmilgravis Hospital, and diagnosed according to the 2010 revised McDonald criteria [52]. The cohort comprised 194 patients with relapsing-remitting MS (mean age: 38.83 ± 9.54 years) and 94 patients with secondary progressive MS (mean age: 50.46 ± 10.24 years). This sample represents 14.18% of the Latvian MS cohort in 2022 (out of 2035 confirmed cases), making it sufficiently representative of the demographic trends in a small population (less than 1.8 million people).

The control group consisted of 253 healthy individuals (148 females, mean age: 46.87 ± 5.26 years; 105 males, mean age: 52.27 ± 8.54 years), recruited from the Latvian Maritime Medicine Centre, Vecmilgravis Hospital (n = 60) and the Genome Database of the Latvian Population, Latvian Biomedical Research and Study Centre (n = 193) (http://biomed.lu.lv/gene/ accessed on 10 September 2021). Control subjects were carefully selected to exclude individuals with autoimmune, cardiovascular, metabolic, or inflammatory diseases. The cohort predominantly represents an admixed population of non-Baltic ethnic groups in Riga, forming a genetic profile typical for north-eastern Europe.

All participants provided written informed consent, and the study was conducted in accordance with the Declaration of Helsinki, with approval from the Central Medical Ethics Committee of Latvia.

### 4.2. DNA Extraction and Genotyping

Genomic DNA was isolated from nucleated blood cells using the GeneJET Genomic DNA Purification Kit (Thermo Fisher Scientific, Waltham, MA, USA) according to the manufacturer’s instructions. Polymerase chain reaction (PCR) was carried out using DreamTaq DNA polymerase (Thermo Fisher Scientific) under the following cycling conditions: initial denaturation at 94 °C for 5 min, followed by 35–40 cycles of 94 °C for 45 s, annealing at 60 °C for 45 s, and elongation at 72 °C for 45 s, with a final extension step at 72 °C for 7 min.

The single nucleotide polymorphisms (SNPs) rs4588 (NG_012837.3:g.57915C>T, chr4; NC_000004.12:g.71752606G>T, GRCh38.p14) and rs7041 (NG_012837.3:g.57904T>G, chr4; NC_000004.12:g.71752617A>C, GRCh38.p14) in the GC gene were identified using restriction fragment length polymorphism (RFLP) analysis. Since these polymorphisms are located 11 base pairs apart, PCR amplification was performed using a single primer pair. Primer sequences were designed using the Primer-BLAST tool (https://www.ncbi.nlm.nih.gov/tools/primer-blast/ accessed on 30 September 2022), with the following sequences: forward primer 5′-GCCTGTGTTCACAGACTCTTTTG-3′ and reverse primer 5′-GGACTTCCAATTCAGCAGCGA-3′. The resulting 634 bp amplicons were subjected to digestion in a reaction volume of 10 µL using restriction enzymes StyI and HaeIII (5 U/μL, Thermo Fisher Scientific, Waltham, MA, USA) to determine genotype variations. DNA quality and concentration were assessed by agarose gel electrophoresis and spectrophotometry. As a quality control measure, 16 randomly chosen samples were genotyped twice in independent experiments, yielding a 100% concordance rate. To further confirm the accuracy of genotyping, selected DNA fragments were sequenced in both forward and reverse directions using the Applied Biosystems 3130xl Genetic Analyzer. SNP nomenclature and nucleotide positioning were assigned following standardized recommendations (https://hgvs-nomenclature.org/stable/ accessed on 30 September 2022).

### 4.3. Measurement of Plasma 25(OH)D Concentration

The concentration of the major circulating vitamin D metabolites (25(OH)D2 and 25(OH)D3) was determined in 131 MS patients using a commercial ELISA kit (IBL International, Hamburg, Germany), which has a detection limit of 11.6 ng/mL, and has a maximum uncertainty of 13.6%. The test results’ absorbance (OD) values were read using a Spark^®^ microplate reader (Tecan, Männedorf, Switzerland) at 450 nm with a reference wavelength of 600–650 nm.

### 4.4. Data Management and Analysis

Single locus genotypes and allele frequencies were estimated by direct gene counting. Deviation from the Hardy–Weinberg equilibrium (HWE) and differences between case and control groups in allele and genotype frequencies were evaluated by χ2 of Fisher exact test using IBM SPSS Statistic v.25 (https://www-01.ibm.com/support/docview.wss?uid=swg21476197/ accessed on 15 September 2023). Different contingency tables were used to design genetic models for the investigated locus, establishing their relationships to the underlying genetic framework. The primary genotyping data were used in the current case–control study to construct multi-locus genotypes and haplotypes and identify the main effects of MS in single and multi-locus models. An odds ratio (OR) of more than two (2) and less than 0.5 was clinically significant [53].

Calculating differences between groups of genotypes by the concentration of the major circulating vitamin D metabolites was performed with Kruskal–Wallis tests.

The results of the comparisons, or *p*-values, were corrected using Bonferroni’s correction for multiple comparisons, and only the corrected results are shown in the tables.

## 5. Conclusions

This study provides novel insights into the association between GC gene polymorphisms (rs7041 and rs4588) and multiple sclerosis (MS) susceptibility in the Latvian population. Our findings indicate that the rare alleles rs7041-T and rs4588-A, along with their corresponding haplotypes, exhibit a protective effect against MS, whereas the common alleles rs7041-G and rs4588-C are associated with increased disease risk. Additionally, while no statistically significant differences were observed in 25(OH)D levels among different VDBP isotypes, a trend emerged, with the Gc1F/1F isotype showing the highest average 25(OH)D levels and Gc1S/2 carriers having the lowest levels.

Study limitations include the potential confounding factors such as drug therapy and vitamin D supplementation, and the lack of additional biochemical markers, such as free 25(OH)D and VDBP concentrations, which could have provided further mechanistic insights. Additionally, environmental and epigenetic factors (e.g., DNA methylation, histone modifications, and miRNA regulation of the GC gene) were not investigated but may play a role in disease susceptibility.

Future research should aim to validate these findings in larger and ethnically diverse cohorts, explore the functional consequences of GC gene polymorphisms on VDBP metabolism, and investigate the impact of epigenetic modifications on vitamin D regulation. Genotype-guided vitamin D supplementation strategies and personalized MS risk assessment models should also be explored to optimize disease prevention and management in high-prevalence populations like Latvia.

## Figures and Tables

**Table 1:** Results of Linkage Disequilibrium analysis of GC gene loci.

Gene Location in the Genome	SNP 1 ID	SNP 2 ID	Distance in the Genome (bp)	LD Analyse					
				MS			Control		
				D′	r^2^	*p*-Value	D′	r^2^	*p*-Value
*GC* chr4:71741693-71805520	rs4588	rs7041	72,618,323–72,618,334	0.99	0.60	<0.00001	0.80	0.39	<0.0001

MS—multiple sclerosis; LD—linkage disequilibrium; D′—normalized frequency deviation. r^2^—correlation coefficient. bp—base pair.

**Table 2:** Distribution of alleles and genotypes of rs4588 and rs7041 polymorphisms and data on association with multiple sclerosis in Latvian population.

*GC* SNPs	MAF/Genotype	Distribution of Genotypes				Data on Association			
		MS (296)		Control (253)		Additive Model		Multiplicative Model	
		N *	%	N *	%	OR_A_ [95% CI]	*p*-Value	OR_M_ [95% CI]	*p*-Value
rs4588	A	178	30.07	192	37.94	0.65 [0.51–0.83]	5.00 × 10^−4^	-	
	CC	136	45.95	91	35.97	reference		1.50 [1.02–2.19]	3.63 × 10^−2^
	CA	142	47.97	132	52.17	0.82 [0.55–1.23]	0.33	1.22 [0.87–1.70]	0.26
	AA	18	6.08	30	11.86	0.39 [0.24–0.64]	2.08 × 10^−4^	0.62 [0.40–0.96]	2.95 × 10^−2^
rs7041	T	244	41.22	259	51.19	0.70 [0.54–0.90]	5.25 × 10^−3^	-	
	GG	94	31.76	60	23.72	reference		1.52 [1.08–2.15]	1.59 × 10^−2^
	GT	160	54.05	123	48.62	0.71 [0.50–1.02]	6.36 × 10^−2^	0.84 [0.60–1.17]	0.31
	TT	42	14.19	68	26.88	0.40 [0.21–0.76]	4.15 × 10^−3^	0.60 [0.32–1.11]	0.10

* The number of patients in multiple sclerosis (MS) and Control groups. The number of genotypes (N) in the groups is equal to the number of samples, the number of alleles is represented by twice the number of genotypes, MAF—minor allele frequency; *p*—Probability calculated by χ2 or Fisher exact tests; OR [95% CI]—odds ratio with 95% confidential interval.

**Table 3:** Data on the association of GC haplotypes, multi-locus genotypes, and isotype/Gc variants in MS and control groups.

Multi- Locus Group *		Alleles/ Genotypes		Distribution of Haplotypes and Genotypes				Statistical Analysis			
				MS		Control		Additive Model		Multiplicative Model	
		rs7041	rs4588	N	%	N	%	OR_A_ [95% CI]	*p*-Value	OR_M_ [95% CI]	*p*-Value
Haplotypes											
H1	1S	G	C	347	58.61	229	45.26	reference		1.72 [1.36–2.19]	7.90 × 10^−6^
H2	1F	T	C	67	11.32	85	16.80	0.52 [0.36–0.74]	3.04 × 10^−4^	0.63 [0.45–0.89]	8.20 × 10^−3^
H3	2	T	A	177	29.90	176	34.78	0.66 [0.51–0.86]	2.23 × 10^−3^	0.80 [0.62–1.03]	*0.078*
H4 ^#^	2	G	A	1	0.17	16	3.16	0.04 [0.01–0.31]	6.80 × 10^−6^	0.05 [0.01–0.39]	6.03 × 10^−5^
DBP(Gc) isotype variations											
G1	1S/2	TG	CA	120	40.54	87	34.66	reference		1.28 [0.90–1.81]	0.17
G2	1S/1S	GG	CC	93	31.42	50	19.92	1.36 [0.88–2.12]	0.17	1.86 [1.25–2.76]	1.88 × 10^−3^
G3	1F/2	TT	CA	21	13.51	36	14.34	0.42 [0.23–0.77]	4.63 × 10^−3^	0.45 [0.26–0.80]	5.46 × 10^−3^
G4	1F/1S	TG	CC	40	13.51	33	13.15	0.88 [0.51–1.50]	0.64	1.03 [0.63–1.69]	0.91
G5	2/2	TT	AA	18	6.08	24	9.56	0.54 [0.28–1.06]	0.072	0.61 [0.32–1.15]	0.12
G6 ^#^	1F/1F	TT	CC	3	1.01	8	3.19	0.27 [0.07–1.05]	0.061	0.31 [0.08–1.18]	0.12
G7 ^#^	1S/2	GG	CA	1	0.34	7	2.79	0.10 [0.01–0.86]	2.27 × 10^−2^	0.12 [0.01–0.96]	2.67 × 10^−2^
G8 ^#^	2/2	TG	AA	-	-	3	1.20	0.58 [0.51–0.65]	4.40 × 10^−2^	0.54 [0.50–0.59]	0.10
G9 ^#^	2/2	GG	AA	-	-	3	1.20	0.58 [0.51–0.65]	4.40 × 10^−2^	0.54 [0.50–0.59]	0.10

MS—multiple sclerosis, *—for haplotype and genotype multi loci form was predicted Gc protein isoform; ^#^—rare haplotype or genotype; N—a number of patients; *p* values according to the χ2 criterion; ORA, ORM is the odds ratio for an additive or multiplicative model; 95% CI—confidence interval.

**Table 4:** Comparison of 25(OH)D levels and multi-locus genotypes and isotype/Gc variants distribution in Multiple Sclerosis cohort.

Genotype ^					25(OH)D Levels, ng/mL								Statistical Analysis
	N, 131	%	Two-Locus Genotype *	Gc Isotype	Average	SD	Min	Max	CI95%		Median	IQR	
G1	59	45.04	TG-CA	Gc1S/2	21.53	6.55	12.20	39.49	19.82	23.24	20.34	10.95	0.26
G2	35	26.72	GG-CC	Gc1S/1S	24.12	7.46	7.37	49.26	21.56	26.68	23.16	8.55	
G3	10	7.63	TT-CA	Gc1F/2	24.48	10.10	11.76	39.89	17.26	31.71	21.32	18.95	
G4	18	13.74	TG-CC	Gc1F/1S	24.20	7.63	14.86	39.52	20.41	27.99	21.30	13.35	
G5	7	5.34	TT-AA	Gc2/2	23.66	5.72	15.11	33.41	18.37	28.96	23.11	6.57	
G6	2	1.53	TT-CC	Gc1F/1F	29.31	0.24	29.14	29.48	27.15	31.47	29.31	0.00	

^—coding of genotypes according to Table 3; * The two-locus genotype is a construct of the rs7041-rs4588 loci; N—the number of patients (in total 131); SD—standard deviation; CI 95%—95% confidential interval of the mean; IQR—interquartile range.

## Data Availability

The data that support the findings of this study are available from the corresponding author upon reasonable request.

## Supplementary Materials

The following supporting information can be downloaded at: https://www.mdpi.com/article/10.3390/ijms26062555/s1.
