# Supplementary material for: Search for Disease-Specific Genetic Markers Originated from the Vitamin D Binding Protein Gene Polymorphisms in the Multiple Sclerosis Cohort in the Latvian Population
Source: Int J Mol Sci. 2025 Mar 12;26(6):2555. doi: 10.3390/ijms26062555 (PMC11941955; doi:10.3390/ijms26062555)
Supplement: Supplementary file 1 [file ijms-26-02555-s001.zip › Supplementary_material.pdf]

Supplementary Material

Table S1 Clinical characteristics of a group of patients with multiple sclerosis (MS)

Clinical characteristic	Patients (N = 288)	
	Average ± SD	Median (IQR)
Data from history of MS		
Age of illness (years) in time of:		
1st symptoms	29.40 ± 9.42	29.00 (14.00)
1st visit and registration of MS	35.73 ± 10.56	36.00 (17.00)
Other autoimmune diseases - yes number (%)	36 (15.19)	
At moments of research		
Disease course, years		
*from 1st symptoms	13.31 ± 9.71	11.00 (12.00)
**from 1st visit	6.97 ± 4.38	6.00 (8.00)
Age (years)	42.70 ± 11.22	43.00 (16.00)
Expanded Disability Status Scale (EDSS; points)	3.76 ± 1.70	3.50 (3.50)
MS phenotype:		
Relapsing-remitting: number (%)	194 (67.36)	
Secondary progressive: number (%)	94 (32.64)	
Laboratory parameters:		
Immunoglobulin G (IgG),	1.65 ± 0.75	1.46 (0.92)
Immunoglobulin A (IgA)	2.19 ± 0.80	1.99 (0.93)
Immunoglobulin M (IgM)	11.64 ± 2.35	11.45 (3.71)
Cluster of differentiation 3 (CD3)	1.43 ± 0.41	1.40 (0.53)
Cluster of differentiation 4 (CD4)	0.90 ± 0.28	0.89 (0.36)
Cluster of differentiation 8 (CD8)	0.54 ± 0.23	0.53 (0.31)
Special medicament therapy - yes number (%)	227 (78.82)	

*From 1st symptom - the duration of time from the registration of the first symptoms in the patient according to the patient's own survey to the control point in time of this study; **from 1st visit - confirmation of the diagnosis during the first visit of the study to the control point in time of this study
